# Healthy Homes

**Published:** 1883-01

**Authors:** 


### HEALTHY HOMES.

Robert Rawlinson, C. E., has given the following admirable rules for securing healthy houses, in his "Letters and Papers on Sanitary Questions."

The following are rules that should be attended to:

The subsoil beneath a house should be naturally dry, or it should be made dry by land draining.

The ground floor of a house should not be below the level of the land, street, or road outside.

A site excavated on the side of a hill, or steep bank, is liable to be dangerous, as external ventilation may be defective, and the subsoil water from above may soak toward and beneath such houses. Middens, ashpits, and cesspools, if at the back, must also taint such basements.

The subsoil within every basement should have a layer of concrete over it, and there should be full ventilation.

Cesspools, cess-pits, sink-holes, or drains, should not be formed nor be retained within house basements.

The ground around dwelling-houses should be paved, flagged, asphalted, covered with concrete, or be graveled.

Outside channels should be in good order, and be regularly cleansed.

House eaves should be guttered and spouted.

Swill tubs should not be near doors or windows.

Pigsties should ever be at a distance; and, where pigs are kept, there should be rigid cleanliness. Improperly keeping pigs has caused more human sickness and destroyed more life than all the battles the country has been ever engaged in.

Garden plats should, of course, be in order, and be property cultivated.

Many houses, from the mansion to the cottage, are unwholesome for some of the following reasons:

1. Damp and unventilated basements.
2. Cesspools and foul drains within the basement.
3. Rotten timber in floors and skirtings and tainted wall papers.
4. Kitchen sinks in improper places and unventilated.
5. Water closets in improper places and unventilated.
6. Rooms without adequate means for ventilation.
7. Water cisterns and pumps in improper places, supplying contaminated water.

These defects should be remedied by landlords. Houses are also unwholesome from accumulated dirt, carelessness, and personal neglect. As when :

1. Rooms are not sufficiently cleansed.
2. Carpets are left down too long and never swept.
3. Windows are seldom opened.
4. Water closets are dirty, neglected, and without ventilation.
5. Dirty beds are unmade and shrouded by dirty hangings.
6. Dirty wardrobes, and dirty clothes' closets.
7. Nooks corners and shelves which are never dusted.

There are points of construction to be attended to which I will name, so as to put them on record for the remembrance of those who may, at some time or other, build cottages or small houses, or be in communication with those who do build, or are going to do so.

Do not build on heaps of rubbish, fillings in with cesspool refuse, chemical waste, or on swampy ground which cannot be drained. Thousands of houses have been so placed, and are now being so placed in the suburbs of our towns.

A bed of concrete over the site of cottages will vastly modify otherwise objectionable positions ; but, indeed, a bed of concrete should be used in all cases.

To ventilate stairs and passages, open the staircase or passage window, or both, by drawing down the top sash several inches in summer, one or more inches in winter, and in some cases screw the sash fast, so that these windows must be open all the year round ; if there is a skylight above the staircase, let there be ventilation here which cannot be closed. The result will be improved health to the family. Pay no attention to any casual remark, " How cold your staircase is !" Let the ladies put on an extra shawl. But the remark will seldom be made.

Schools, as a rule, are very defectively ventilated, ordinary flat-ceilinged rooms are totally unfit for public schools. The space should be open up to the roof-ridge, and this should be louvered.

Nurseries and children's rooms should be permanently ventilated. Dormitories for children should have ample ventilation ; clothe the children warmly, cover the beds warmly, prevent direct draughts, and the cool air will not injure.

Avoid flue ventilation of every sort ; let the fresh air come in as direct as possible. Night air is the only air you can have at night, so do not fear it. Dread foul, because tainted, air manufactured within the rooms. Any outside fresh air is better than lung and skin tainted inside air.

